# Is there an association between consent rates in Swiss hospitals and critical care staffs' attitudes towards organ donation, their knowledge and confidence in the donation process?

**DOI:** 10.1371/journal.pone.0211614

**Published:** 2019-02-08

**Authors:** Isabelle Keel, Roger Schürch, Julius Weiss, Marcel Zwahlen, Franz F. Immer, Comité National du Don d’Organes (CNDO)

**Affiliations:** 1 Swisstransplant, the Swiss National Foundation for Organ Donation and Transplantation, Bern, Switzerland; 2 CTU Bern, Department of Clinical Research and Institute of Social and Preventive Medicine (ISPM), University of Bern, Bern, Switzerland; 3 Institute of Social and Preventive Medicine (ISPM), University of Bern, Bern, Switzerland; Indiana University, UNITED STATES

## Abstract

This study investigated the critical care staff’s attitude, knowledge and involvement with donation, skills and confidence with donation-related tasks and their association with consent rates at the hospital level. In 2015, we conducted a cross-sectional survey among critical care staff of hospitals involved in organ donation using an anonymous online questionnaire with a response rate of 56.4% (n = 2799). The hospital level consent rate was obtained from the Swiss Monitoring of Potential Donors database (2013–2015). For each hospital, we calculated a mean score for each predictor of interest of the Hospital Attitude Survey and investigated the association with hospital consent rates with generalized linear mixed-effect models. In univariable analysis, one score point increase in doctors' confidence resulted in a 66% (95% CI: 45%–80%) reduction in the odds to consent, and one score point increase in nurses' attitudes resulted in a 223% (95% CI: 84%–472%) increase in the odds to consent. After simultaneously adjusting for all major predictors found in the crude models, only levels of education of medical and nursing staff remained as significant predictors for hospital consent rates. In Switzerland, efforts are needed to increase consent rates for organ donation and should concentrate on continuous support as well as specific training of the hospital staff involved in the donation process.

## Introduction

Organ shortage is a global problem [1]. Switzerland has had a low post mortem donation rate compared to other developed countries for over a decade [2]. In the meantime the demand for organ transplantation is steadily increasing and there is a chronic imbalance between the number of donors and the number of patients on the waiting lists.

A low donation rate may be due to a variety of factors. The attitude, knowledge, skills and educational needs of the critical care (CC) staff is one element that may influence donation rates and which has not been evaluated in Switzerland since 2008 [3]. What several studies show is that among the general population the overall support of organ donation is high, but decreases if the questions get more personal (Would you donate your own organs? Would you consent to organ donation of a family member?). Furthermore, to achieve donation, more education is needed for each step of the donation process [3–6]. In a study by Roels at al. the results of the Hospital Attitude Survey from 11 countries were compared and correlated with the donation performance of each country. It was confirmed that there is a link between the attitude of the CC staff, acceptance of the brain death concept and confidence in the subject of organ donation and a successful conversion from potential to actual donors [3].

Numerous measures were implemented in Switzerland to increase the number of organs available for transplantation [7–9]. Six networks have been created and each hospital is affiliated to one of them with the aim to support smaller detection hospitals regarding organ donation. Regular communication trainings are offered on how to talk with the next of kin about organ donation and obtaining consent [9]. The Swiss Donation Pathway [10] gives recommendations on how to handle each step in the donation process. Each hospital has a local donor coordinator who is responsible for all organ donation related tasks. A quality assurance tool (Swiss Monitoring of Potential Donors—SwissPOD) has been developed with the aim to provide detailed information on the detection and referral of potential organ donors. SwissPOD tries to monitor reasons for non-donation, and to identify means to increase the rates of donation in the future [10]. Irrespective of all measures taken, donation rates remain at the same level (around 13 donors per million of population) and consent rates are very low (less than 50%). The low consent rate in Switzerland seems to be one of the biggest factors of losing potential for organ donation [11]. Several studies investigated predictors of consent. Higher consent rates were associated with receiving understandable information, the timing of the request and the person making the request. Also, health care staff who attended education programs regarding organ donation achieved higher donation rates [11–14].

Clearly donation rates depend on various factors [3–5,8,13,15–20]. Switzerland has an opt-in organ donation policy (explicit consent). Therefore obtaining consent is a necessary condition for organ donation. There is some evidence that the attitude and knowledge of the (CC) staff has an effect on donor management and on the family consent rate [3,13]. In Switzerland the legal regulations (Transplantation Ordinance, Chapter 8 Section 1. Art.45ff) require the following: potential organ donors have to be identified, brain death diagnosis must be performed and the next of kin have to be informed and consent for donation has to be obtained from the next of kin [21]. Therefore the CC staff’s participation is crucial in the organ donation process.

This study aimed to investigate the critical care staff's attitude, knowledge and involvement with donation, skills and how comfortable they feel with donation-related tasks and if there is a correlation with consent rates.

## Materials and methods

### Survey / Datasets

This study was a cross-sectional survey conducted with an anonymous online questionnaire in 2015. The survey aimed at assessing self-reported CC staffs’ attitudes and knowledge regarding organ donation. The questions were mainly close-ended questions with the exception of two open-ended questions at the end where participants were asked what should be done to improve organ donation in their opinion. The responses to the open-ended questions are not analyzed here. Participation in the survey was voluntary.

To assess the attitude, knowledge, skills, and educational needs of the CC staff, we used a simple 42-item anonymous online questionnaire (www.surveymonkey.com). The questions were based on the Hospital Attitude Survey of the Donor Action Program and were translated to three national languages spoken in Switzerland (German, French, Italian). The questions were slightly adjusted to the Swiss situation. The questions were split in 5 sections: 1. Staff attitudes to organ donation, 2. Involvement in the donation Process, 3. Skills/self- confidence levels, 4. Knowledge of in-hospital processes regarding organ donation, 5. Educational needs in donation issues.

In addition, demographic data such as gender, age, function and position, years of experience in the current workplace, and birth canton or country (if not Switzerland) was asked.

To correlate the results of the Hospital Attitude Survey with consent rates, we used data from SwissPOD from 2013–2015. SwissPOD is a national quality assurance tool that started in 2011 aiming at providing detailed information on the detection and referral of potential organ donors to assure quality in the donation process as stipulated by the law. Each death in intensive care units (ICU) and accident & emergency departments (A&E) is entered into the SwissPOD database. Data is only entered by trained staff and each case is validated by data monitors at Swisstransplant to check the plausibility and assure data quality. We merged the SwissPOD data to the hospital attitude survey data using hospital identifiers.

The protocol of our study (including the survey questionnaire) has been reviewed and approved by the Review Board of the Comité National du Don d’Organes (CNDO; principal investigator of the study). The review and approval of the study protocol includes warranting that it is in full compliance with applicable Swiss law which requires neither review nor approval by an ethics committee for studies that include no patient data at all. Survey participants were health care professionals exclusively, and participation in the survey was voluntary and anonymous. The authors had no access to participant identifying information as part of this work.

### Participants and setting

The study sites included all 72 Swiss hospitals with an ICU recognized by the Swiss Society of Intensive care (SGI). Eligible for participation in the survey were all members of the CC staff (nurses and doctors of ICU and A&E departments), and also neurologists and neurosurgeons of the participating hospitals as they are often involved in brain death diagnosis. A link to the anonymous questionnaire was provided to the communication departments of the hospitals or, in hospitals without a communication department, to the local coordinator for organ donation. The communication departments and local donor coordinators then distributed the link to the questionnaire to the eligible participants and reported back to us how many links were sent to be able to calculate a response rate. In total, 4965 links were sent and 2799 questionnaires were received (response rate of 56.4%).

### Primary outcome

The primary outcome is the consent rate: The consent rate for each hospital is calculated from the number of cases where consent to organ donation was given divided by the total number or cases where permission for donation was sought. SwissPOD will give the number of possible/potential organ donors where permission for donation was sought and the total number of obtained consents for donation per hospital for the years 2013–2015. Hospitals where no approach in view of seeking permission for donation happened are excluded as they do not have an outcome. Seeking permission for donation almost exclusively happens on the ICU. Therefore, the A&E will be excluded in this analysis.

### Predictors of interest

For each of the predictors of interest in which the response was favorable, we built a score per dimension and the mean was calculated for each hospital (Table 1). The Dimensions of interest are:

- Attitude
- Knowledge
- Confidence
- Involvement
- Education

**Table 1:** Each question relevant to build the score.

Dimension	Answers considered favorable	Score
Attitude: What is your general attitude to donation of organs and tissues for transplants? Would you donate some of your organs after death? Brain death is a valid determination of death	yes or yes with restrictions	0–3
Knowledge: Does your hospital have standardized procedures concerning the donation process? Does your hospital have formal guidelines for obtaining consent for organ donation?	yes	0–2
Confidence: Do you feel comfortable in explaining brain death to the next of kin? Do you feel comfortable in obtaining consent for organ donation? Do you feel comfortable in comforting / supporting grieving families?	yes	0–3
Involvement: Number of cases you were involved in the last calendar year in communicating information on severe brain damage to next of kin? Number of cases you were involved in the last calendar year in explaining brain death to next of kin? Number of cases you were involved in the last calendar year in obtaining consent for organ donation? When would you consider the most appropriate time to bring up the subject of organ donation to the next of kin?	all except for none	none = 0 1–3 = 2 4–6 = 5 > 6 = 8
Education: Have you received education in brain death concept? Have you received education in communication skills (including grief counselling) in the donation process?	yes	0–2

The predictors of interest were chosen and limited as not all questions in the questionnaire are relevant concerning the consent rate. Specifically, questions as from the stage in the donation process where organ donation is a real possibility were selected. This means an interaction with the next of kin regarding organ donation is a subject. The questions in the Dimension "Attitude" were selected as there is some evidence that the attitude of the critical care staff has an effect on the family consent rate [3,13].

### Statistical analysis

The statistical analysis comprised two parts. In the first part, the results of the cross-sectional survey were analyzed and the responses to the questions as asked described. Variability of answers were assessed according to professional category and type of medical care unit. The consent rate is calculated on the hospital level. To check for a correlation we had to calculate a mean score for each predictor of interest on a hospital level. Each answer to the questions of interest resulted in a defined score. The scores were added up and a mean was calculated. Then we compared regional differences in attitudes using Kruskal-Wallis test, followed by Tukey post-hoc comparisons.

In the second part, we analyzed the association between consent rate at the hospital level and attitudes towards organ donation of the critical care staff, their knowledge and confidence in the donation process and their educational needs. This analysis was done in two ways. First consent rate at the hospital level and summaries of the survey responses (mean scores per hospital, separately for different professional categories (doctors vs. nurses)) at the hospital level was calculated and their association investigated using grouped logistic regression models. Each variable was evaluated individually at the beginning and we present crude odds ratios (OR) and corresponding 95% confidence intervals (CI).Predictive variables with good association (p < 0.2) were used for multivariate analysis with multiple variables, to identify independent correlation factors of the interest variables. In a last analysis, the hospitals were nested within networks and the data was analyzed using binomial generalized linear mixed models allowing each network its own intercept. Statistical significance was assessed using likelihood-ratio tests.

A 2-sided p-value of < 0.05 is considered statistically significant. The statistical analysis was performed with R.

## Results

### Survey results

**Fig 1:**
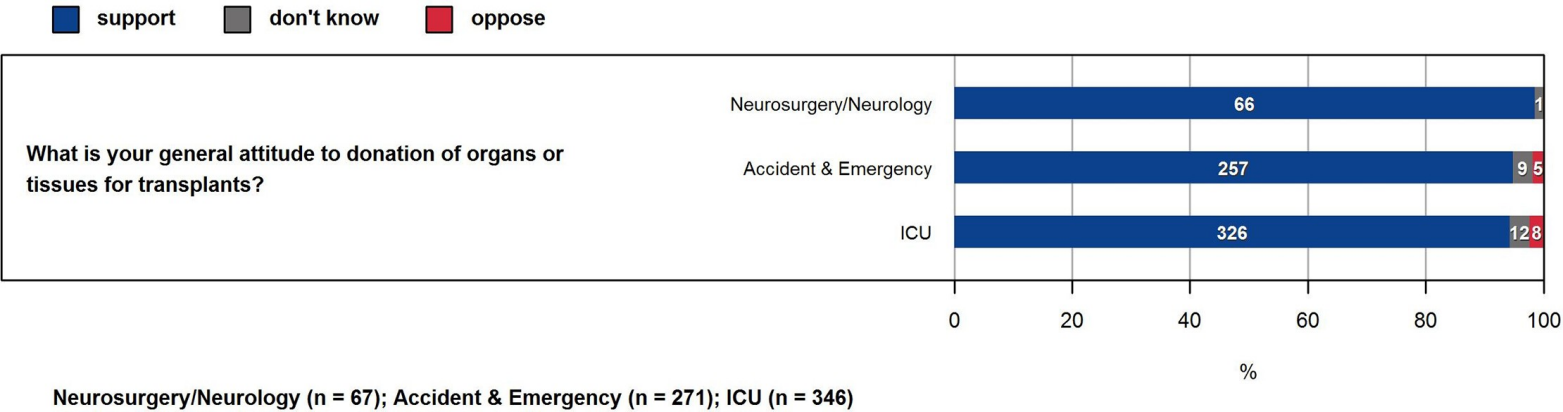
Attitude of medical staff towards organ donation. Nursing staff is similar (not shown).

**Fig 2:**
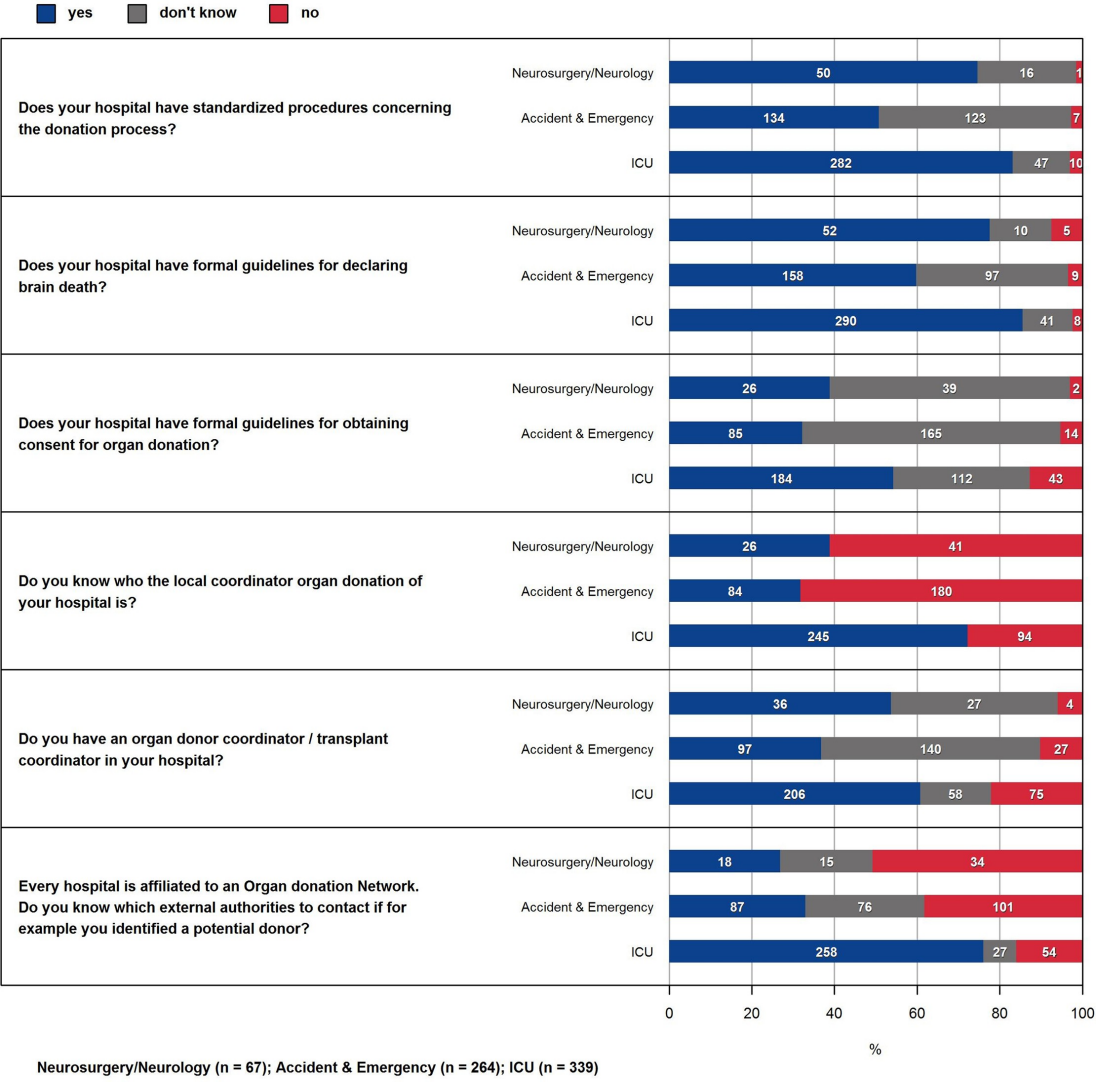
Knowledge of medical staff about donation process in the hospital. Nursing staff is similar (not shown).

**Fig 3:**
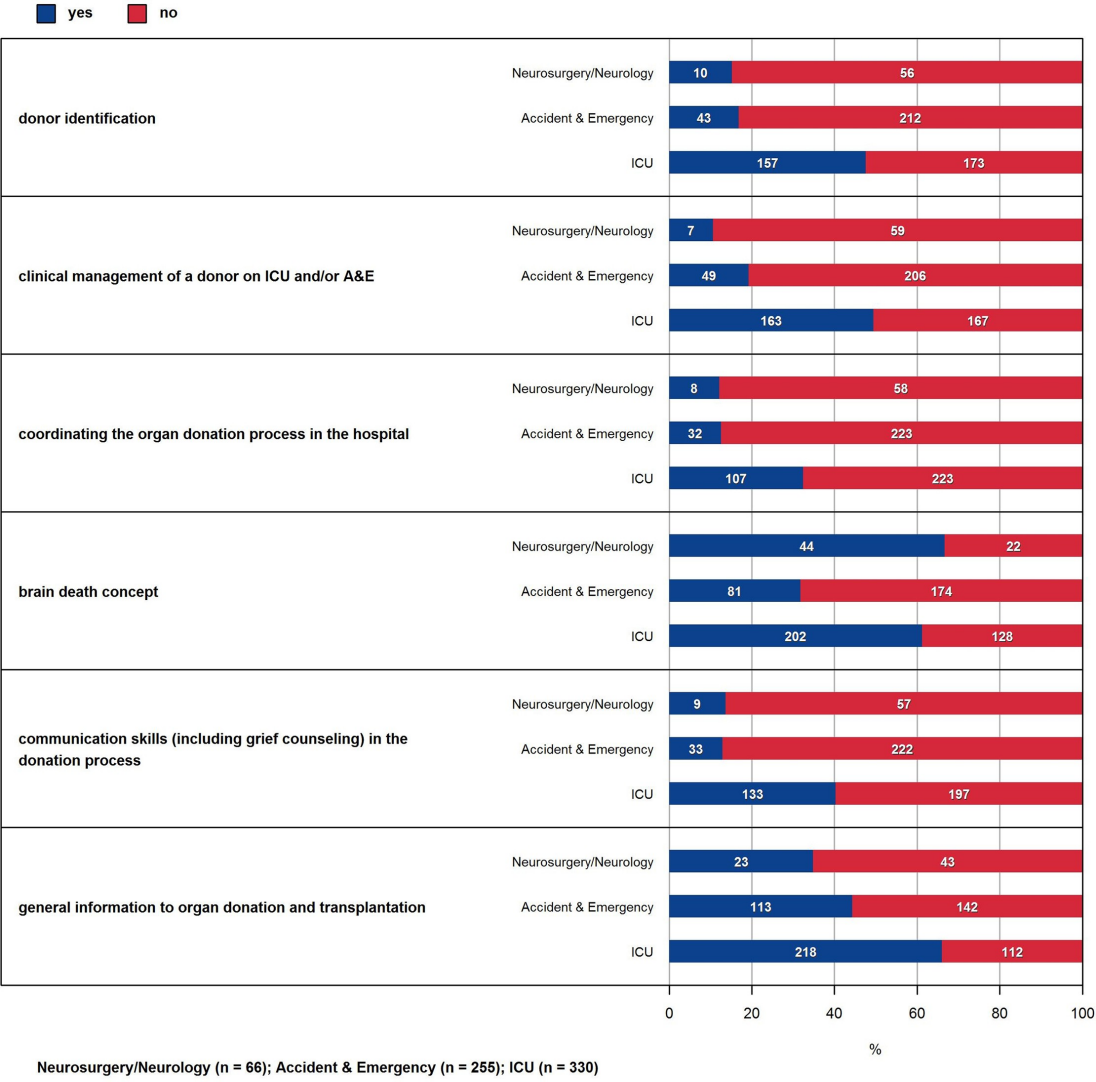
Education of medical staff in regard to organ donation. ‘Yes‘ means that respondents received training. Nursing is staff similar (not shown).

In total, 2799 participants of the 72 hospitals the questionnaire was sent to completed the survey. Of these, 705 (25.2%) were medical staff and 2094 (74.8%) nursing staff. The highest number of respondents was specialized nurses with 44.6% and of the medical staff, senior residents with 11%. 59.3% of all respondents work on an ICU. Majority of respondents were female (71.1%). The characteristics of the survey participants are given in Table 2. For all of Switzerland, attitudes towards organ donation is overwhelmingly positive (Fig 1), whereas nursing and medical staff seem to lack knowledge regarding the process of donation (Fig 2, Fig 3).

**Table 2:** Participant characteristics for the Hospital Attitude Survey.

	n	%
**Professional category** (N = 2799)		
Medical Staff	705	25.2
Nursing Staff	2094	74.8
**Function** (N = 2799)		
Chief physician	64	2.3
Resident / Registrar (senior)	308	11.0
Resident (junior)	265	9.5
Physician—Other	66	2.4
Head Nurse	108	3.9
Specialized nurse	1249	44.6
Registered nurse	558	19.9
Student Nurse	24	0.9
Assistant Nurse	36	1.3
Nurse—Other	121	4.3
**Medical Care Unit** (N = 2799)		
ICU	1659	59.3
Accident & Emergency	1053	37.6
Neurosurgery/Neurology	87	3.1
**Sex** (N = 2488)		
male	718	28.9
female	1770	71.1
**Age** (N = 2488)		
18–24	41	1.6
25–34	902	36.3
35–44	850	34.2
45–54	522	21.0
>55	173	7.0
**Years worked in specialty** (N = 2487)		
<1	134	5.4
1 to 5	598	24.0
6 to 10	606	24.4
11 to 20	692	27.8
>20	457	18.4

### Regional differences in scores

**Fig 4:**
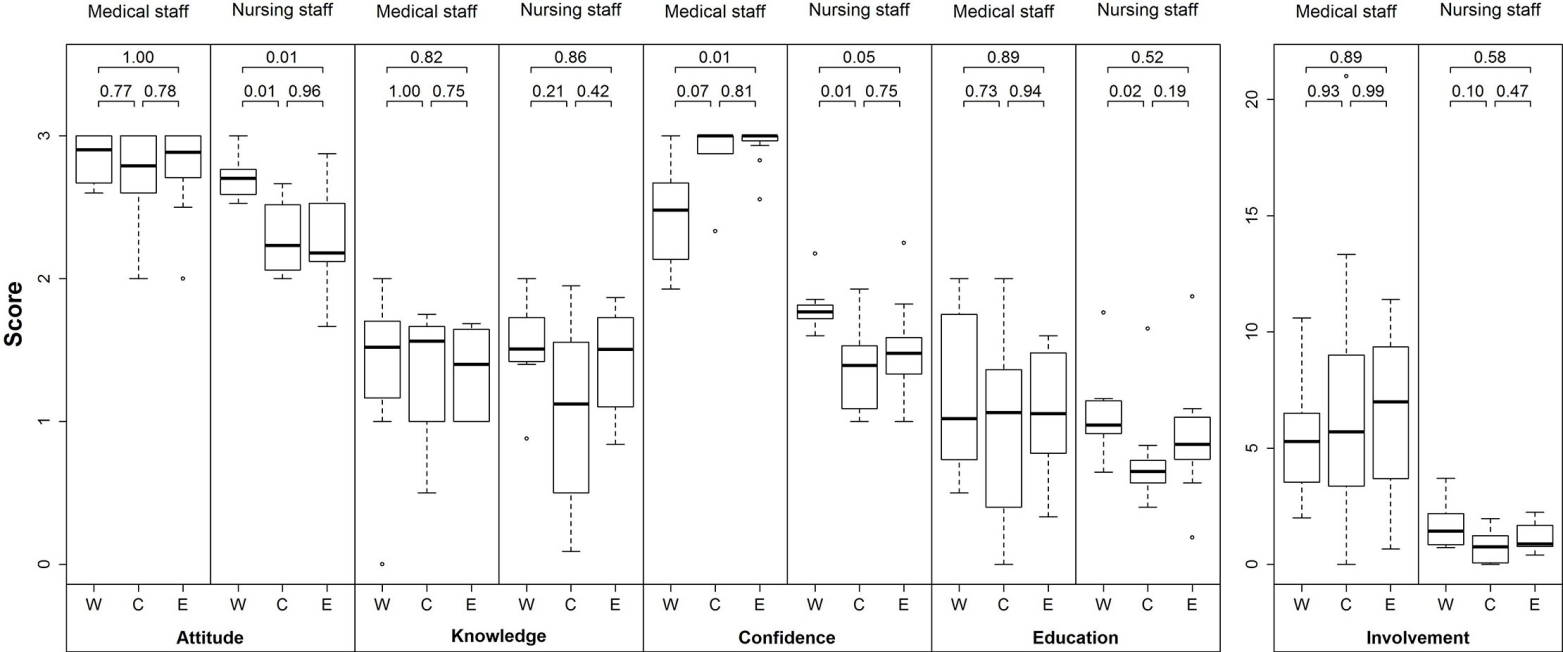
Regional differences in scores. W = Western, C = Central, E = Eastern Switzerland. Boxes represent interquartile range, whiskers extend to the extreme values or to 1.5 times the interquartile range. p-values stem from post-hoc Tukey tests after group comparison with Kruskal-Wallis test.

As from this point of the analysis only hospitals which have at least one next of kin approach are included. Due to the fact that certain networks only have one hospital in which the next of kin was approached to seek consent for donation we split Switzerland in three regions. West (8 hospitals of the French and Italian speaking part of Switzerland). Central (10 hospitals from the networks Bern, Basel and Lucerne) and East (12 hospitals from the networks DCA and St. Gall). For each of the predictors of interest, we built a score per dimension (attitude, knowledge, confidence, involvement, education) and the mean was calculated for each hospital. After aggregating survey results into scores we found considerable differences among the regions (Fig 4). In particular, we found that attitude of nursing staff in Western Switzerland differed from that of nurses in Central and Eastern Switzerland, and that confidence of medical and nursing staff in Western Switzerland differed from confidence in Central and Eastern Switzerland.

### Association between consent rates and survey scores

**Fig 5:**
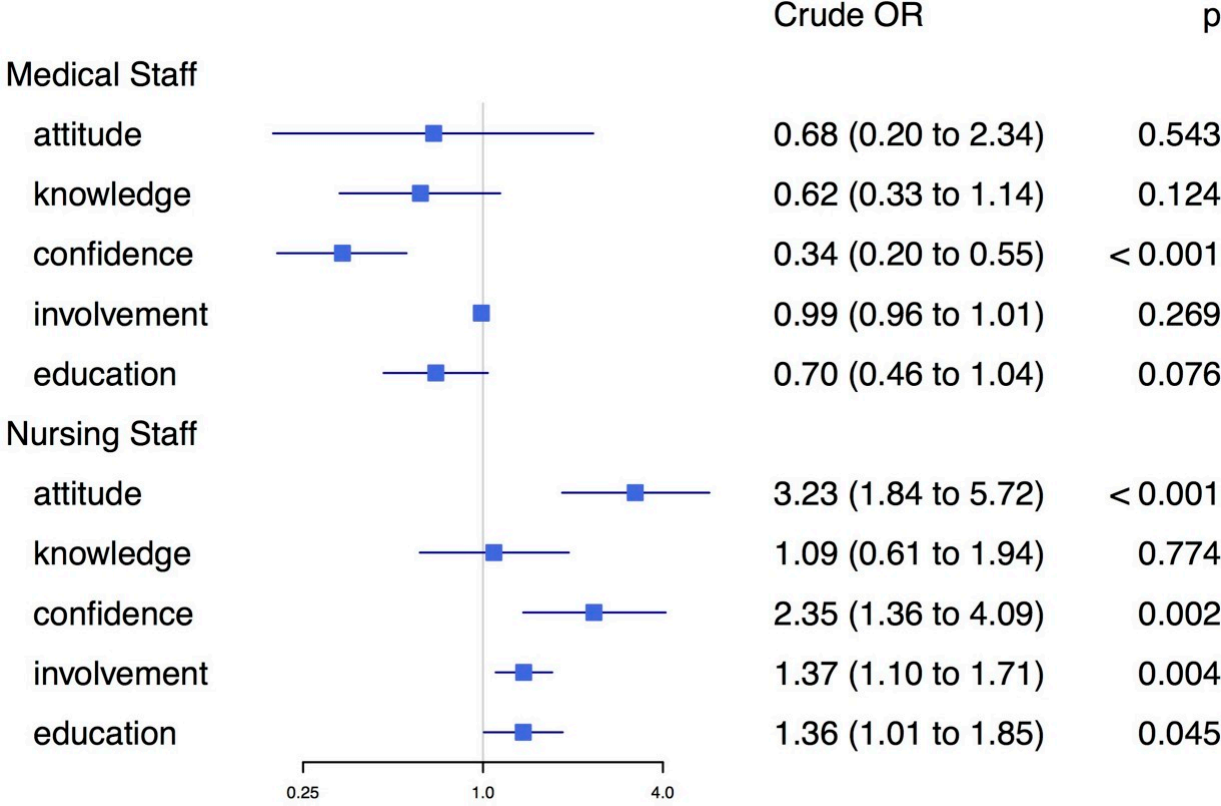
Forest plot presenting univariate odds ratios for predictors of interest.

The crude analysis of the association between donation consent rates and survey scores identified knowledge, confidence, and education of medical staff (doctors) as influential predictors of consent rate (Fig 5). In addition, attitude, confidence, involvement, and education scores of nursing staff showed a strong association with consent rate (Fig 5). Confidence of medical staff and attitudes of nursing staff were the strongest single predictors of consent rate, where a score point increase in doctors' confidence resulted in approx. 66% reduction in the odds to consent, and where a score point increase in nurses' attitudes resulted in approx. 223% increase in the odds to consent.

After simultaneously adjusting for all major predictors found in the crude models, we found that only education of medical and nursing staff remained as significant predictors (Table 3).

**Table 3:** Adjusted model.

	fully adjusted model	
	OR (95% CI)	p
**Medical Staff**		
knowledge	1.34 (0.53 to 3.42)	0.540
confidence	0.52 (0.25 to 1.09)	0.082
education	0.38 (0.17 to 0.83)	0.016
**Nursing Staff**		
attitude	1.51 (0.60 to 3.81)	0.383
confidence	0.48 (0.11 to 2.08)	0.327
involvement	1.60 (0.98 to 2.64)	0.060
education	2.06 (1.22 to 3.50)	0.006

**Fig 6:**
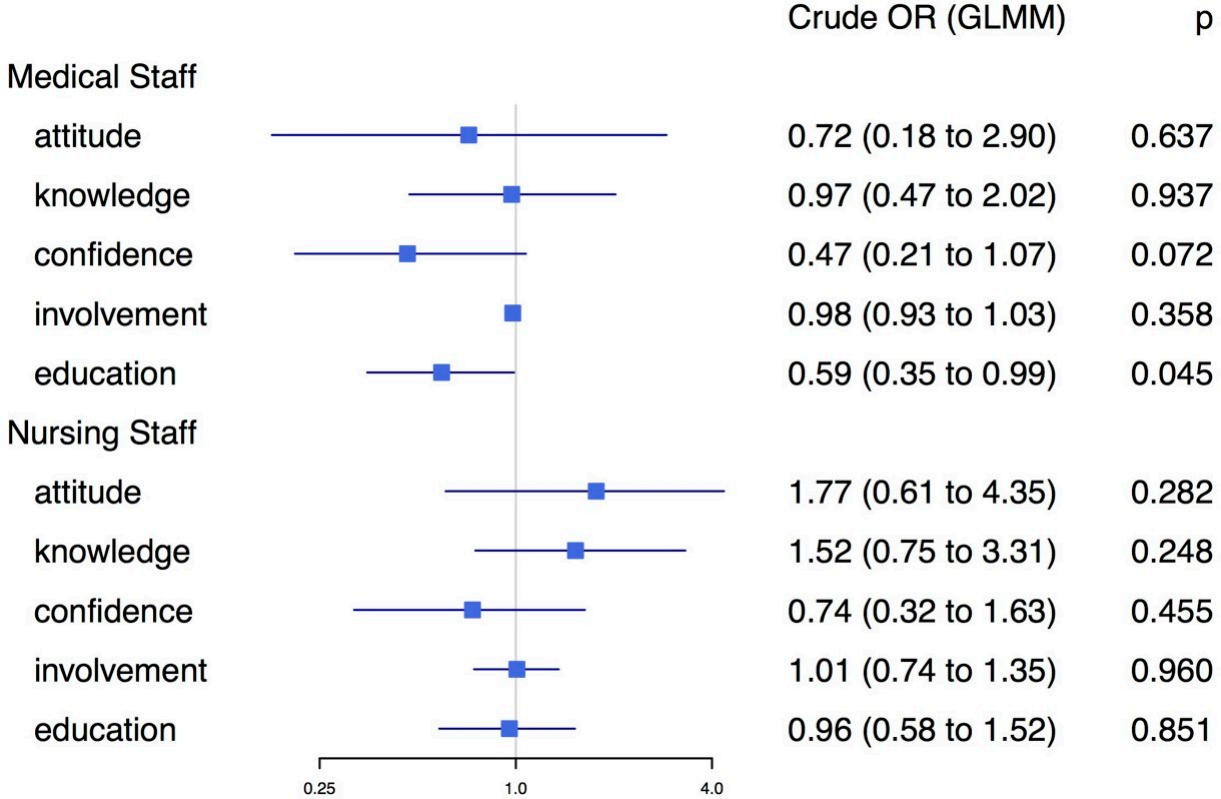
Forest plot presenting univariate odds ratios from a GLMM for predictors of interest, allowing each network its own intercept.

The strong co-linearity among the predictors of interest as well as the instability of the adjusted model, suggest that there might be inherent differences among regions and networks (see Fig 4). To adjust for such differences among networks, we used generalized linear mixed-effect models (GLMM) to allow each network its own intercept. After allowing each network its own intercept, none of the predictors were significant (Fig 6).

## Discussion

This is the first study in Switzerland that tried to investigate an association between consent rates to organ donation and attitude, knowledge, confidence, education and involvement of the CC staff in organ donation related tasks. In the large Hospital Survey with over 2799 respondents we found that a very large majority of respondents are supportive towards organ donation. There were differences in confidence levels in performing organ donation related tasks among nurses and doctors. Overall the results indicate that more education is needed.

In the crude analysis we found the attitude of nurses and confidence of doctors to be the strongest predictors of consent. The attitude of nurses was positively associated with the consent rate, whereas the consent rate was lower when doctors scored high in confidence. However, after adjusting the crude model, only education remained a significant predictor of consent. After looking at the regions, we found that none of the predictors of interest were significant, which shows the strong co-linearity among the predictors of interest and the differences among regions.

In the Swiss population a large majority is supportive towards organ donation [22]. Therefore, the favorable view of organ donation of the respondents was no surprise (Fig 1). Our findings regarding the attitude of the CC staff are in line with other studies, which show an overall very positive attitude towards organ donation among health care professionals [5]. Surprising was, that irrespective of all measures taken, only around 50% of the CC staff know, whether formal guidelines for obtaining consent for organ donation exist in the hospital they are employed at (Fig 2). There seems to be a lack of education for both medical and nursing staff. Only around 40% of the medical and nursing staff had training in communication skills (including grief counselling) in the donation process. Also, only 60% of medical staff and 50% of nursing staff had some kind of training concerning the concept of brain death (Fig 3). Another study that performed a similar cross-sectional survey showed that there is considerable need for more education and training among hospital staff, especially on how to inform and support the donor relatives [4].

As the results showed no significance when looking at the mixed models, we explored the difference by region by looking at the predictors of interest only (Fig 4). Several studies have shown that there are regional differences in Switzerland, and that the attitude towards organ donation among the general population is more positive in the French and Italian speaking parts [11,23,24]. This is also reflected in the findings of our study where nursing staff in the western region seems to be more favorable towards organ donation than nursing staff from the central and eastern regions. Surprisingly, the medical staff of the western region seems to be more self-critical as seen in a lower confidence score than the doctors from the central and eastern regions. Conversely, nurses in the western region had a higher confidence score compared with nurses in the central and eastern regions. This may be due to the fact that the nurses in the western region have been more involved in the donation process than in the central and eastern part. In the western region, mostly nurses fill the position as local donor coordinators, whereas in the central and eastern part it is mainly doctors.

The crude analysis showed a significantly higher consent rate if the nurses' attitudes are positive towards organ donation. The nurses are the specialists that are with the family a lot more than doctors. Therefore, the nursing staff is closer to the family and a nurse might be an important person for giving more information or supporting them in this difficult situation. A study from Spain points out that the attitude of nurses is fundamental in the context of organ donation [25]. It is so, as the authors suggest, because nurses with a negative attitude towards organ donation can influence people who are exposed to the subject, or generate distrust in the process [25]. Another study states that nurses who have a positive attitude have a better practice when it comes to organ donation related tasks [26].

A surprising result in our study was the significantly lower consent rate if the doctors have a high confidence score. This may be explained that they can seem too professional (and therefore somehow impersonal) in such an emotional moment which might appear as arrogant to a grieving family. It may be assumed, however, that the next of kin would need an empathetic person who takes the time to explain them what is happening. Explaining brain death and obtaining consent for organ donation is in most cases not an easy conversation. It is a consistent finding in several studies that a sensitive and empathetic way during discussions with the family leads to higher consent rates [12,27,28]. A systematic review investigated modifiable factors influencing relatives' decision to offer organ donation. It showed that including specialized coordinators from an organ procurement center resulted in a much higher consent rate than when hospital staff approached the next of kin alone [13]. However, other studies point out that obtaining consent is not only influenced by the person who seeks permission for donation, but also the timing of the approach, being given enough time to decide, perceived care in general and if the information they were given is understandable [11–13,29]. In Switzerland, there are guidelines with recommendations as from which time point in the process the next of kin should be approached, or when the transplant/donor coordinator gets involved, but there are no clear regulations. It is usually the treating doctor who decides when specialized staff is called in. Switzerland also does not have transplant/donor coordinators available in every hospital.

In the adjusted model, only education remained as a significant predictor of consent in both, medical and nursing staff. This comes as little surprise as generally it may be assumed that more education entails better knowledge. In addition, the confidence of those who have received specific training might grow, and they may be more involved in the donation process. Education has been investigated in the study of Roels et al. in 2010 [3]. Switzerland participated in that study and it was found that educational needs were high. The need for education is still considerable. Communication courses have been offered for many years, but the blended learning (a combination of e-learning modules and formal education lessons to each step of the donation process) was implemented in mid-2015 only. Therefore, not many have profited from that offer at the point of the study. Another problem might be to find the time for the training. A Polish study has investigated if the implementation of a long-term, homogenous education program (European Training Program on Organ Donation, ETPOD) has an effect on donation and consent rates. They found that in hospitals involved in the ETPOD program, the increase in organ donation was greater than in the hospitals who did not participate in the program. Moreover, a pronounced benefit in obtaining consent for organ donation was observed [30].

A major limitation in our study was that the survey was on a voluntary basis. Looking at the response rate the likelihood is high that mostly hospital staff with a prior interest and a positive attitude towards the subject of organ donation answered the questionnaire. Moreover, as it was self-reported, especially in the dimension of knowledge, we only know the perceived knowledge of the respondents, rather than their actual knowledge in the donation process. A general limitation is that previous analyses of SwissPOD data have shown that the consent rates in hospitals in the German speaking part of Switzerland tend to be lower compared with the French and Italian regions [11,31]. Assuming that obtaining consent depends on several factors (that may also be influencing each other), it is difficult to answer the question of which factor is most decisive in a general manner. Also, it is hard to quantify and weigh the impact of each factor against the others. For example, if a patient or the next of kin are against organ donation, the attitude (even if most positive), knowledge and skills of the hospital staff will have very little influence on that decision. Conversely, if the wish of the patient is unknown and the next of kin are undecided, it seems plausible that their decision may in part or even largely depend on their perception of the staffs’ attitudes towards organ donation. In addition, one can assume that to some degree, also the inverse holds: If the hospital staff is aware of a generally positive and supportive attitude towards organ donation in the population, they will likely be more confident and/or positive when approaching the family. This, in turn, might have a considerable effect on the decision making of the next of kin as they may feel receiving a stronger professional support in this difficult situation.

Therefore, we conclude that efforts in the domain of obtaining consent to organ donation should concentrate on two main areas. First, continuous support as well as specific training of the hospital staff involved in the donation process. This will ensure that they have high levels of confidence and practical knowledge in all the parts of the process they are involved in. Second and equally important, one should make sure that the positive attitude among the population better translates into actual organ donors. This, however, seems not to depend foremost on the attitude and knowledge of the hospital staff, but whether people are willing to take a decision if they would eventually like to be an organ donor or not. Such a decision (which should be documented or at least communicated to the next of kin) would provide more security and ease to both the hospital staff and the next of kin, as they could be certain about the patient's wish. Further direction could include qualitative studies, for example interviews with the next of kin to find out what motivated them to consent to organ donation or what the reasons were for objection to donation. Moreover, the blended learning possibly should be mandatory for at least specialized critical care nurses and medical staff as from level senior resident to ensure overall good knowledge in organ donation related tasks. Another option could be that the organ donation process would be implemented in the curriculum of nursing and medical students.

## Supporting information

S1 File Survey raw data. (XLSX)

S2 File Consent data. (XLSX)
